# The Effect of Plasticizers on the Polypyrrole-Poly(vinyl alcohol)-Based Conducting Polymer Electrolyte and Its Application in Semi-Transparent Dye-Sensitized Solar Cells

**DOI:** 10.3390/membranes11100791

**Published:** 2021-10-18

**Authors:** KM Manikandan, Arunagiri Yelilarasi, SS Saravanakumar, Raed H. Althomali, Anish Khan, Khamael M. Abualnaja, Dalal Alhashmialameer, MA Hussein

**Affiliations:** 1 Department of Physics, Kamaraj College of Engineering and Technology, K.Vellakulam, Virudhunagar, Madurai P.O. Box 625 701, Tamil Nadu, India; yeliloct1@gmail.com; 2 Department of Mechanical Engineering, Kamaraj College of Engineering and Technology, K.Vellakulam, Virudhunagar, Madurai P.O. Box 625 701, Tamil Nadu, India; sankarameena@yahoo.co.in; 3 Chemistry Department, Faculty of Science, King Abdulaziz University, P.O. Box 80203, Jeddah 21589, Saudi Arabia; r.h-t@hotmail.com (R.H.A.); maabdo@kau.edu.sa (M.A.H.); 4 Center of Excellence for Advanced Materials Research, King Abdulaziz University, P.O. Box 80203, Jeddah 21589, Saudi Arabia; anishkhan97@gmail.com; 5 Department of Chemistry, College of Science, Taif University, P.O. Box 11099, Taif 21944, Saudi Arabia; k.ala@tu.edu.sa (K.M.A.); Dsamer@tu.edu.sa (D.A.); 6 Chemistry Department, Faculty of Science, Assiut University, Assiut 71516, Egypt

**Keywords:** plasticizer, dye-sensitized solar cells (DSSC), conducting polymer electrolyte, crystallinity, polypyrrole

## Abstract

In this work, the quasi-solid-state polymer electrolyte containing poly(vinyl alcohol)-polypyrrole as a polymer host, potassium iodide (KI), iodine (I_2_), and different plasticizers (EC, PC, GBL, and DBP) was successfully prepared via the solution casting technique. Fourier transform infrared spectroscopy (FTIR) was used to analyze the interaction between the polymer and the plasticizer. X-ray diffraction confirmed the reduction of crystallinity in the polymer electrolyte by plasticizer doping. The ethylene carbonate-based polymer electrolyte showed maximum electrical conductivity of 0.496 S cm^−1^. The lowest activation energy of 0.863 kJ mol^−1^ was obtained for the EC-doped polymer electrolyte. The lowest charge transfer resistance *R*_ct1_ was due to a faster charge transfer at the counter electrode/electrolyte interface. The polymer electrolyte containing the EC plasticizer exhibited an average roughness of 23.918 nm. A photo-conversion efficiency of 4.19% was recorded in the DSSC with the EC-doped polymer electrolyte under the illumination of 100 mWcm^−2^.

## 1. Introduction

Nowadays, electricity is essential in the society because of the fast-growing industrialization and for domestic applications. Solar radiation is one of the most promising future renewable energy resources for a wide range of applications due to its abundance and easy accessibility and the fact that it does not cause pollution. The solar photovoltaic (PV) technology contributes to generating electricity. Solar cells are applied in charging portable devices, outdoor lightings, electronic signboards on the road, fountain pumps, electric vehicles, agricultural machines, remote communication for the military, etc. Dye-sensitized solar cells (DSSCs) have been developed due to their low cost, semi-transparency, various colors, flexible nature, and simple fabrication process compared to silicon solar cells [1,2,3]. A DSSC comprises a photoanode, a redox electrolyte, and a counter electrode. The use of plasticizers in polymer electrolytes enhance the performance, flexibility, and long-term stability of various electrochemical devices such as dye-sensitized solar cells, fuel cells, batteries, biosensors, chemical sensors, and super-capacitors [4,5].

Plasticizers make polymer electrolytes more flexible, compatible and enhance the mobility of polymeric chains. Plasticizers easily diffuse within polymers and cause polymer deformation and coalescence into a homogeneous film. The main role of plasticizers is to improve the electrical properties of polymers by increasing their electrical conductivity. Ethylene Carbonate (EC), Propylene Carbonate (PC), γ-Butyrolactone or gamma-Butyrolactone (GBL), and Dibutyl Phthalate (DBP) are commonly used plasticizers due to their low viscosity and high dielectric constant [6]. Plasticizers usually possess relatively long alkyl chains and a reduced intermolecular friction between their molecules. It is interesting that the degree of crystallinity of a polymer system shows a decreasing trend when increasing the concentration of the plasticizer. Kesavan et al. reported that the ethylene carbonate-based poly(vinyl pyrrolidone) polymer electrolyte showed maximum ionic conductivity [7]. The ionic conductivity of an un-plasticized polymer electrolyte decreases when a large quantity of salt leads to ion-pair formation and cross-linking, reducing the segmental movement of the polymer chain [8]. A critical problem when using un-plasticized polymer-based electrolytes in DSSCs is due to minimum ionic diffusion, high interfacial charge transfer resistance at the electrolyte/electrode interface, and high surface tension of the polymer electrolyte inside a porous nano-TiO_2_ anode [9]. Ren et al. reported the use of a plasticized gel polymer electrolyte for DSSC with an overall power conversion efficiency of 2.9% [10]. Nogueria et al. reported the addition of plasticizers to a polymer electrolyte led to the highest diffusion coefficient [11]. Ito et al. reported that the addition of γ-Butyrolactone as a plasticizer in P(EO-EPI)/LiI/I_2_ with different content of MMT clay allowed achieving the highest photo-conversion efficiency of 3% [12].

In this study, different plasticizers ((EC, PC, GBL, and DBP) were added to the PVA/PPy/KI/I_2_ polymer electrolyte. The plasticized polymer electrolytes were used in DSSCs and characterized by FTIR, X-ray diffraction, electrical conductivity, EIS, AFM, and photovoltaic measurements. 

## 2. Experimental

### 2.1. Materials 

Polypyrrole (PPy) was purchased from Sigma-Aldrich (St. Louis, MO, USA). Poly(vinyl alcohol) (PVA) was purchased from Nice chemical (Kochi, India). Ethanol, Potassium iodide (KI), and Iodine (I_2_) were procured from Himedia (Mumbai, India). Dimethyl sulfoxide (DMSO) was supplied by Spectrum chemical (New Brunswick, NJ, USA). Triton-X 100 (Iso-octylphenoxy polyethoxyethanol), Ethylene Carbonate (EC), Propylene Carbonate (PC), γ-Butyrolactone or gamma-Butyrolactone (GBL), and Dibutyl phthalate (DBP) were obtained from Loba Chemie (Mumbai, India). Titanium dioxide (TiO_2_) nano powder (P25) was purchased from Degussa (Frankfurt, Germany). Fluorine-doped tin oxide (FTO) glass (~7 Ω/sq) was purchased from Sigma-Aldrich (St. Louis, MO, USA). Platinum catalyst solution (Platisol) and N719 dye [*cis*-diisothio cyanato–*bis* (2,2-bipyridyl-4,4-dicarboxylato) ruthenium (II) *bis*-(tetrabutylammonium)] were purchased from Solaronix (Aubonne, Switzerland). 

### 2.2. Preparation of the Plasticized Polymer Electrolyte 

**Figure 1:**
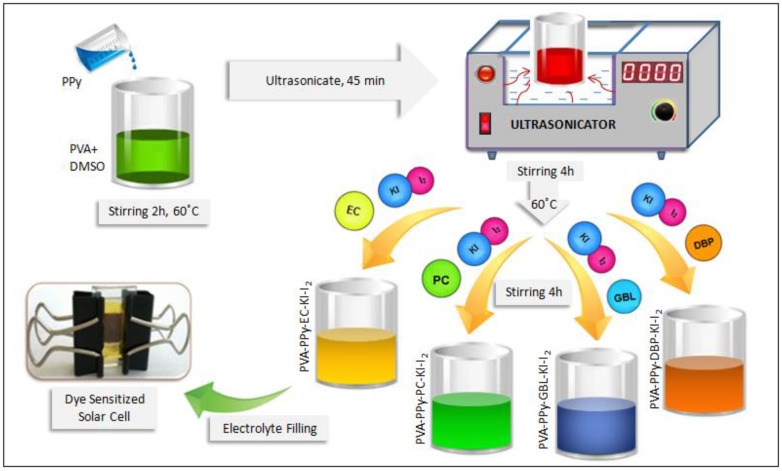
Preparation of the quasi-solid-state conducting polymer electrolyte with different plasticizers. (90 [85 PVA: 10PPy: 5KI:I_2_]:10x, where x = EC, PC, GBL, and DBP).

The quasi-solid-state plasticized polymer electrolyte was prepared by the dissolution of PVA in 10 mL DMSO solvent, stirring continuously for 2 h at 60 °C. Then, 10 wt.% of PPy was added to the PVA solution, which was ultrasonicated for 45 min [13]. In addition, 10 wt.% EC was added to the prepared homogeneous polymer solution. This experiment was repeated using different plasticizers such as PC, GBL, and DBP. The mixture was then stirred for 4 h to achieve complete dissolution and homogeneity of the electrolyte. A redox electrolyte consisting of 0.5 M of KI and 0.05 M of I_2_ was added to the homogeneous solution, which was then stirred continuously for 4 h. Finally, a DSSC was fabricated using the plasticized polymer electrolyte sandwiched between the photoanode and the counter electrode. The preparation of the quasi-solid-state conducting polymer electrolyte with different plasticizers is shown in Figure 1.

### 2.3. Characterization and Measurements

Complex formation between the polymer and the plasticizer was tested using a Perkin Elmer Spectrum Version 10.03.09 FTIR spectrometer (Waltham, MA, USA) at wavelength between 400 and 4000 cm^−1^. 

The crystal structure was determined by using a PANalytical X’Pert PRO powder X-ray Diffractometer (Malvern Panalytical, Malvern, UK) with a scanning rate of 2 deg min^−1^. The degree of crystallinity of the sample was deconvoluted using the original software [6].

*Χ*_c_ = (*I*_C_/*I*_T_) × 100% (1)

*I*_C_, Area under the crystalline peaks and *I*_T_, total area under the diffractogram.

The electrical conductivity (*σ*) of the samples was calculated using a four-probe setup (DEP-02 model),

*σ* = (1/*ρ*) Scm^−1^ (2)

*ρ*, Corrected resistivity (*ρ =ρ_ο_/G7_W/S_); ρ*_ο_, Resistivity (*ρ*_ο_ = *V*/*I* × 2π*S*); *G7*_W/S_, Correction factor*; S*, Probe spacing; *W*, Thickness of the sample; *V*, Voltage; *I*, Current.

Arrhenius plot [ln(z) versus (1/*T*)] allowed the determination of the activation energy can from the slope and intercept of the best-fit line through the data [14]. 

The absorption spectra of the different polymer electrolytes were obtained with a Shimadzu Model UV-1601 scanning double beam UV–Visible spectrophotometer (Shimadzu, Kyoto, Japan). The indirect band gap (*E*_g_) values were determined from (*αhυ*)^1/2^ versus (*hυ*) plots. The absorption coefficient of amorphous materials was related to incident photon energy using the equation

(*αhυ*) = *β*(*hυ* − *E*_g_)^n^ for (*hυ* > *E*_g_) (3)

α = (2.303*A*/*t*) and *A* = log(*I*/*I*_0_); *t*, Thickness of the film; *I*, Transmission intensity; *I*_0_, Incident light intensity; *β*, Constant.

The exponent ‘*n*’ takes the values of ½ and 2 for indirect and direct electron transition, respectively [15].

Electrochemical impedance spectroscopy (EIS) was carried out using an electrochemical analyzer (CH Instrument, Austin, TX, USA)

J–V characteristics under illumination of 100 mW cm^−2^ were determined using a solar simulator (300 W xenon lamp source, Oriel, Orlando, FL, USA) with a Keithley electrometer. The fill factor (*FF*) and photovoltaic conversion efficiency *η* were determined using the following equations:

Fill factor, *FF* = (*V*_max_ × *J*_max_)/(*V*_oc_ × *J*_sc_) (4)

Photovoltaic conversion efficiency, *η* = (*V*_max_ × *J*_max_)/(*P*_in_) × 100% (5)

*J*_sc_, Short circuit current density (mA cm^−2^); *V*_oc_, Open circuit voltage (V); *V*_max_, Maximum voltage (V); *J*_max_, Maximum current density (mA cm^−2^) and *P*_in_, Incident light power (mW cm^−2^).

The surface morphology of the polymer electrolyte films was captured with an atomic force microscope (AFM, Park XE-70 and XEI image processing software, Park Systems, Suwon, Korea).

## 3. Results and Discussion 

### 3.1. Fourier-Transform Infrared Spectroscopy (FTIR) Analysis 

**Figure 2:**
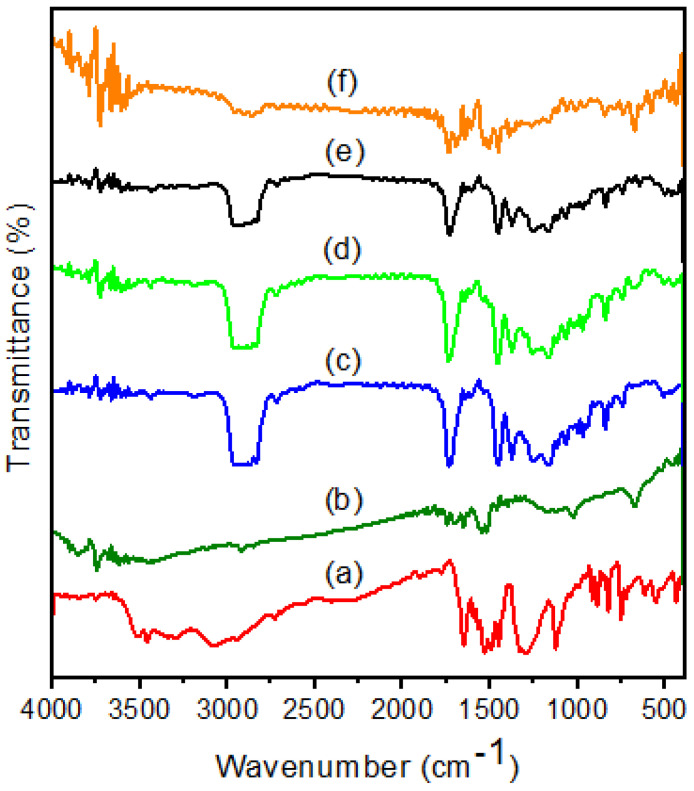
FTIR spectra of (**a**) pure PVA, (**b**) PPy, and PVA/PPy/KI/I_2_ electrolytes with various plasticizers (in 10 wt.%); (**c**) EC, (**d**) PC, (**e**) GBL, and (**f**) DBP.

The interaction between the polymer and the plasticizer in the electrolyte system was analyzed by FTIR spectroscopy. Changes in the molecular vibration modes were due to the interaction between the polymer and the plasticizer. The FTIR spectra of pure PVA, pure PPy, and PVA/PPy/KI/I_2_ with different plasticizers (EC, PC, GBL, and DBP) are shown in Figure 2. The wide-ranging band observed at 3600–3200 cm^−1^ corresponds to the –OH group in PVA. The characteristic peak at 1731 cm^−1^ was ascribed to the C=O stretching of PVA. The peak at 1629 cm^−1^ corresponds to the C=C stretching of the pyrrole ring. The peaks at 1183 cm^−1^ [16] and 1130 cm^−1^ represent the C–N stretching vibration of the PPy ring and the C–H bending of PPy. The peak at 1564 cm^−1^ corresponds to N–H bending vibrations of the PPy ring [17,18]. The peaks at 1623, 1399, and 1123 cm^−1^ were ascribed to pure KI salt [19]. The new peak at 1159 cm^−1^ corresponds to the C–O–C stretching of the ethylene carbonate (EC) plasticizer in the polymer electrolyte [20]. The peak at 1454 cm^−1^ was attributed to −CH_3_ asymmetric bending vibrations of PC [21]. The peaks at 1767 and 1036 cm^−1^ correspond to the –C=O (carbonyl) stretching mode and the –C–O stretching of pure GBL [22].

The peaks at 2946 and 2879, that at 1600, and that at 1076 cm^−1^ were assigned to C–H, C=C, and C–O stretching for pure DBP, respectively. The peaks at 1159 cm^−1^ of EC, 1454 cm^−1^ of PC, 1767 and 1036 cm^−1^ of GBL, and 2946, 2879, 1600, and 1076 cm^−1^ of DBP were found to be shifted in the FTIR spectra of their respective complexes. The vibrational peaks of PVA and PPy were slightly shifted, and this was observed for all the polymer complexes. Moreover, the position of the peak shifted to a certain extent, which confirmed that a strong interaction occurred between the plasticizer and the PVA/PPy/KI/I_2_ electrolyte.

### 3.2. X-ray Diffraction Analysis 

**Figure 3:**
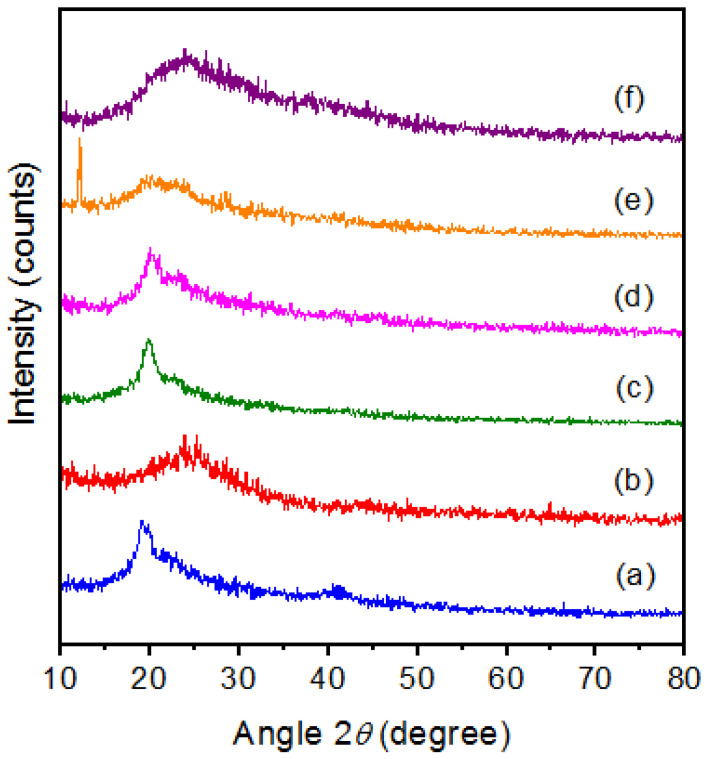
XRD patterns of (**a**) pure PVA, (**b**) PPy, and PVA/PPy/KI/I_2_ electrolytes with various plasticizers (in wt.%); (**c**) EC, (**d**) PC, (**e**) GBL, and (**f**) DBP.

Figure 3 shows that the X-ray diffraction patterns of (a) pure PVA, (b) pure PPy, and PVA/PPy/KI/I_2_ electrolytes with various plasticizers (in wt.%), i.e., (c) EC, (d) PC, (e) GBL, and (f) DBP. The diffraction peak found at 19.8° indicates that semi-crystalline structure of pure PVA. A broad diffraction peak between 20° and 30° indicates the amorphous nature of pure PPy. The degree of crystallinity of the prepared plasticized polymer electrolytes were 9.75% for EC, 10.66 % for PC, 10.35% for GBL, and 9.99% for DBP. The obtained results showed that the degree of crystallinity of the polymer electrolytes varied with the addition of the plasticizers. Additionally, the EC-based polymer electrolyte had the lowest degree of crystallinity, as shown in Table 1. It can be noticed that the incorporation of the EC plasticizer into the PVA/PPy polymer composite matrix led to the separation of the polymer chains, followed by a rearrangement of the structure and a reduction of the viscosity of the polymer electrolyte [23].

### 3.3. UV–Vis Spectra Analysis 

**Figure 4:**
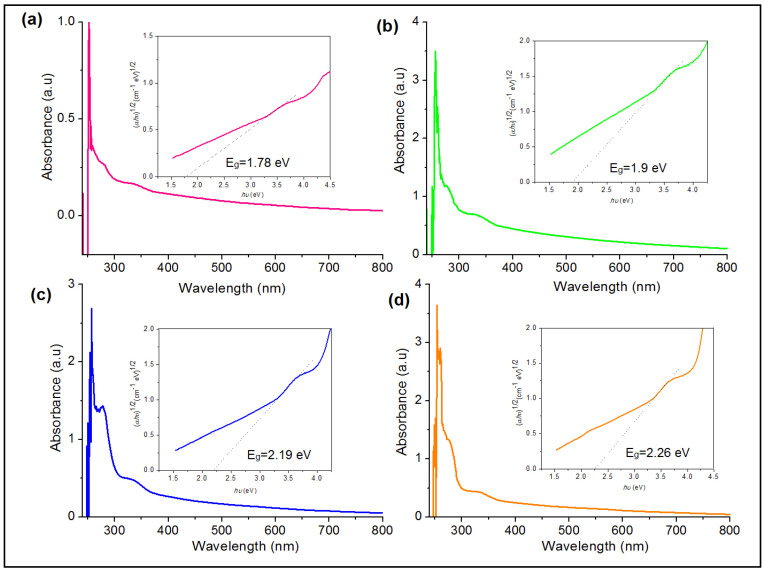
Absorption spectra of PVA/PPy/KI/I_2_ electrolytes with various plasticizers (in wt.%); (**a**) EC, (**b**) PC, (**c**) GBL, and (**d**) DBP. The inset shows the indirect band gap.

Figure 4 shows the absorption spectra of the PVA/PPy/KI/I_2_ electrolyte with various plasticizers (in wt.%), i.e., (a) EC, (b) PC, (c) GBL, and (d) DBP. The fundamental absorption peak appearing around 250 nm for all plasticized polymer electrolytes is due to the π–π* transition [24,25]. The intensity change of the absorption bands reflecting the variation of the band gap energy is due to the crystallinity of the plasticized polymer electrolytes [26]. The indirect band gap values of the plasticized polymer electrolytes EC, PC, GBL, and DBP were 1.78, 1.90, 2.19, and 2.26 eV, respectively. The EC-based polymer electrolyte system showed the lowest band gap value compared to the other system, as summarized in Table 2. The *E*_g_ value decreased from 2.14 eV for the un-plasticized polymer electrolyte to 1.78 eV for the EC-based PVA/PPy/KI/I_2_ electrolyte. The lowest *E*_g_ value indicates the change in the energy states of the conduction band and valence band, which led to a slight shift of the electronic structure of the polymer matrix [27]. 

### 3.4. Electrical Conductivity Measurement

**Figure 5:**
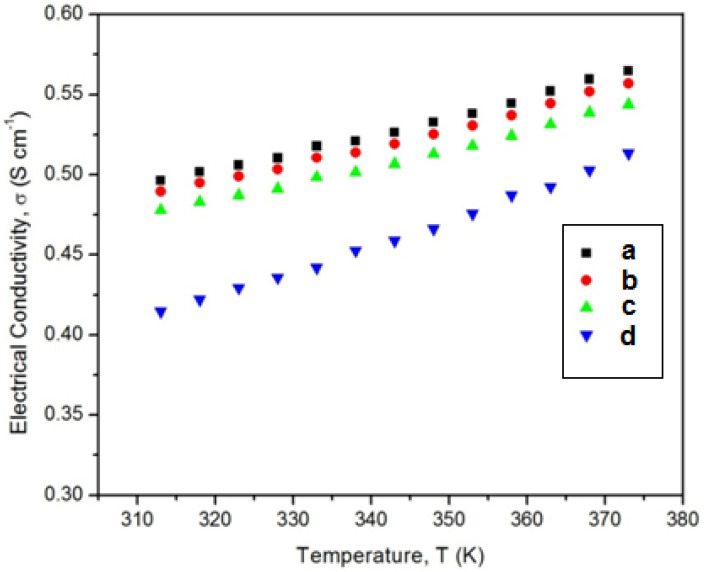
Electrical conductivity–temperature plots of the PVA/PPy/KI/I_2_ electrolyte with various plasticizers (in wt.%); (**a**) EC, (**b**) PC, (**c**) GBL, and (**d**) DBP.

**Figure 6:**
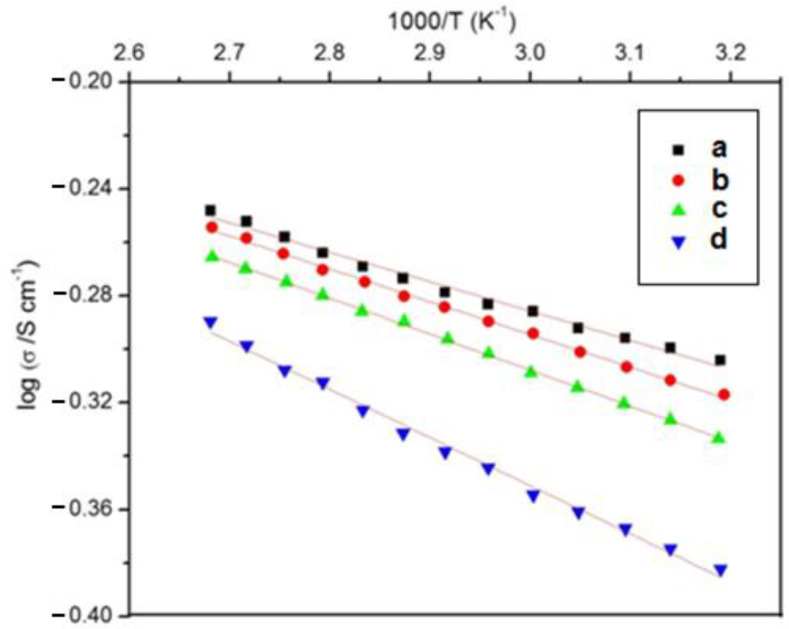
Plots of log *σ* versus 1000/*T* for the PVA/PPy/KI/I_2_ electrolyte with various plasticizers (in wt.%); (**a**) EC, (**b**) PC, (**c**) GBL, and (**d**) DBP.

Figure 5 shows the variation of the electrical conductivity with different temperatures of the PVA/PPy/KI/I_2_ electrolytes containing various plasticizers. It is clearly indicated that the PVA/PPy/KI/I_2_ electrolyte containing EC exhibited the maximum electrical conductivity of 0.496 S cm^−1^ at 313K, which is relatively higher compared to that of the other plasticized polymer electrolytes (Table 3). It was noticed that, as the temperature increased, the electric conductivity also increased for all plasticized polymer electrolytes, which was due to a low charge transfer resistance. Thus, the electrical conductivity of the EC-based polymer electrolyte increased due to fast oxidation and reduction processes that took place in the polymer electrolyte. The *E*_a_ values for all plasticized polymer electrolytes were analyzed using the Arrhenius plot (Figure 6). The plasticized polymer electrolyte containing EC showed the lowest value of *E*_a_ as compared to the other polymer electrolytes containing PC, GBL, and DBP. Therefore, EC-based polymer electrolytes with low activation energy are desirable to obtain highly efficient DSSCs [28]. The calculated *E*_a_ values of all plasticized polymer electrolytes are summarized in Table 3. 

### 3.5. Electrochemical Impedance Spectroscopy Analysis

**Figure 7:**
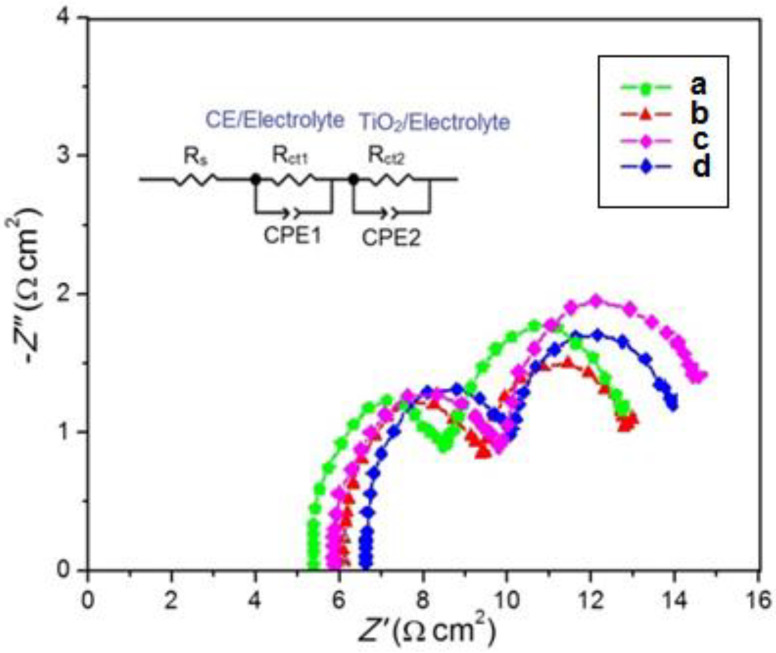
Nyquist plots of the PVA/PPy/KI/I_2_ electrolyte with various plasticizers (in wt.%); (**a**) EC, (**b**) PC, (**c**) GBL, and (**d**) DBP.

Figure 7 present the Nyquist plot of EIS spectra and shows the DSSC filled with the PVA/PPy/KI/I_2_ electrolyte with various plasticizers. Two semicircles are present in the Nyquist plot. The intersection with the horizontal axis in the high-frequency region indicates the series resistance (*R*_s_). A semi-circle in the high-frequency and low-frequency regions corresponds to the charge transfer resistance at the counter electrode/electrolyte interface, i.e., *R*_ct1_, and at the photoanode/electrolyte interface, i.e., *R*_ct2_. The EC-based plasticized polymer electrolyte showed a smaller charge transfer resistance *R*_ct1_ than the other plasticized polymer electrolytes containing PC, GBL, and DBP. This result indicated a faster charge transport at the interface between the platinum-coated counter electrode and the plasticized polymer electrolyte [25]. Additionally, the charge transfer resistance *R*_ct2_ of the EC-based plasticized polymer electrolyte system was 3.879 Ω cm^2^, which was smaller than those of the other plasticized polymer electrolytes (Table 4). This was due to the low charge transfer resistance between the polymer electrolytes and the photoanode. As a result, a decreased recombination rate at the electrolyte/electrode interface [29] and a faster electron transport mechanism were confirmed in the DSSC with the EC-based polymer electrolyte. The lowest series resistance *R*_s_ observed in the EC-based polymer electrolyte may contribute to an enhanced conduction due to a higher electrical conductivity [30]. 

### 3.6. Photovoltaic Performances

**Figure 8:**
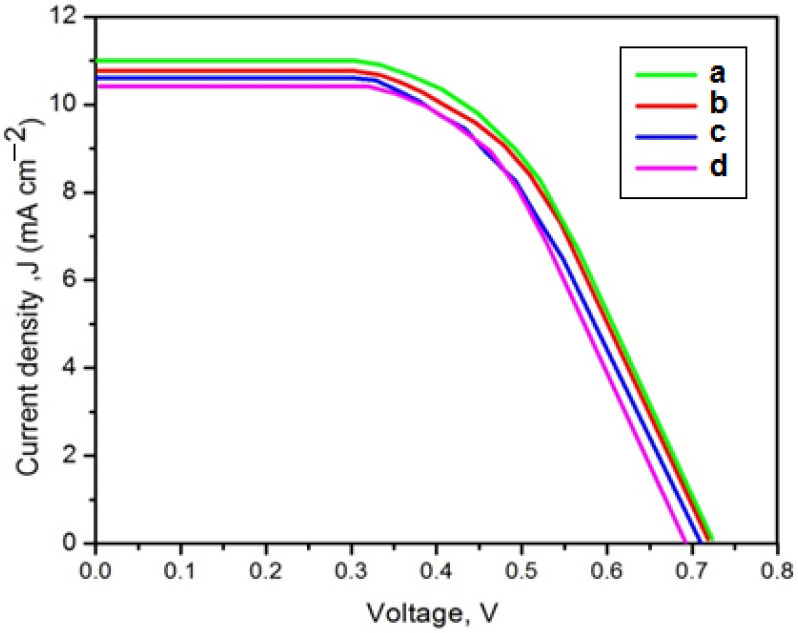
Photo current density (*J*)–voltage (*V*) curves for the DSSCs fabricated with the PVA/PPy/KI/I_2_ electrolyte containing various plasticizers (in wt.%); (**a**) EC, (**b**) PC, (**c**) GBL, and (**d**) DBP.

The *J–V* characteristics of the four dye-sensitized solar cells are presented in Figure 8. The calculated photovoltaic parameters are summarized in Table 5. The DSSC using the EC-based polymer electrolyte achieved the maximum photo-conversion efficiency of 4.19% under illumination of 100 mW cm^−2^. Moreover, the minimum interfacial resistance value attributed to the increased contact area at the polymer electrolyte/photoanode and electrolyte/counter electrode interfaces in the EC-based polymer electrolyte led to an increase in the short-circuit current density [31]. The higher *J_sc_* value indicated the acceleration of charge carriers in the EC-based polymer electrolyte [32]. Moreover, the open circuit voltage (*V*_oc_) of all plasticized polymer electrolytes was almost equal, indicating that the device did not depend on the different plasticizers.

### 3.7. AFM Analysis

**Figure 9:**
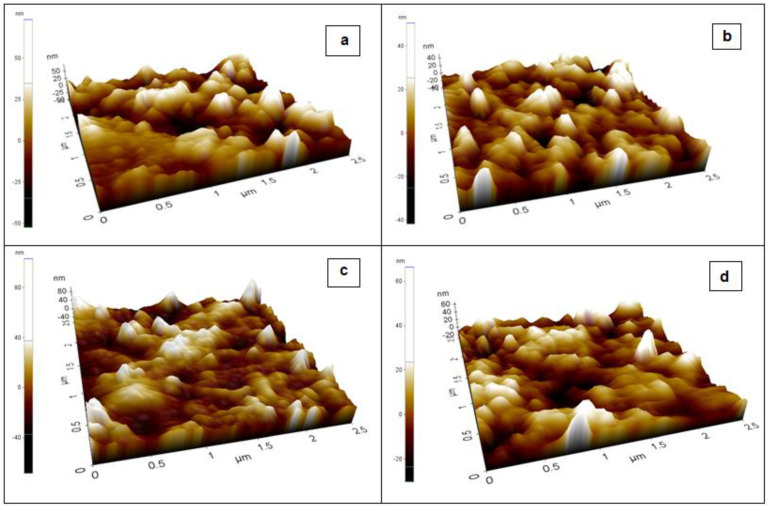
Image showing the 3-D surface roughness of the PVA/PPy/KI/I_2_ electrolyte with various plasticizers (in wt.%); (**a**) EC, (**b**) PC, (**c**) GBL, and (**d**) DBP.

Figure 9 shows the AFM 3D topographical images of the PVA/PPy/KI/I_2_ electrolyte with various plasticizers. The average roughness *R*_a_ of EC is 23.918 nm, smaller than those of PC, GBL, and DBP, which are 28.724, 38.437, and 30.012 nm, respectively. In addition, the roughness skewness *R*_sk_ of EC is −0.185 nm, higher than those of the other plasticized polymer electrolytes. The EC-based polymer electrolyte exhibited the higher negative value of *R*_sk_ due to its porous nature, which is desirable for entrapping the electrolyte. The roughness kurtosis *R*_ku_ values for EC, PC, and DBP are 2.67, 2.237, and 2.985 nm, respectively. The *R*_ku_ value of GBL is 4.199 nm, which indicates a spiky surface; for all the other samples it is less than 3 nm, which indicates a rough surface. The EC-based polymer electrolyte showed the lowest *R*_rms_ compared to the other plasticized samples. Finally, a low *R*_a_ may increase the surface contact at the plasticized polymer electrolyte/electrode interface [13].

## 4. Conclusions 

Quasi-solid-state polymer electrolytes with various plasticizers were prepared by the solution casting process. The interactions between polymers, salt, and different plasticizers were confirmed by FTIR. The degree of crystallinity of the plasticized polymer electrolytes was determined by XRD. The polymer electrolyte containing EC exhibited the highest electrical conductivity of 0.496 Scm^−1^. The EC-based polymer electrolyte exhibited lower activation energy as well as reduced bandgap energy compared to the other plasticized polymer electrolytes. From the EIS analysis, the charge transfer resistances *R*_ct1_ and *R*_ct2_ at the polymer electrolyte/electrode interface of the EC-based polymer electrolyte was significantly smaller than those of the other plasticized polymer electrolytes. The EC-based polymer electrolyte exhibited a higher negative value of *R*_sk_ due to its porous nature, which is a desirable feature for entrapping the electrolyte. The photo-conversion efficiency of 4.19% was recorded in the DSSC with the EC-doped polymer electrolyte under the illumination of 100 mWcm^−2^. 

## Figures and Tables

**Table 1:** Degree of crystallinity of the PVA/PPy/KI/I_2_ electrolyte with different plasticizers.

Sample	*I*_T_	*I*_C_	Crystallinity, *χ* (%)
PVA/PPy/EC/KI/I_2_	2530.14	246.68	9.75
PVA/PPy/PC/KI/I_2_	2353.80	251.13	10.66
PVA/PPy/GBL/KI/I_2_	2586.73	267.96	10.35
PVA/PPy/DBP/KI/I_2_	2561.90	255.84	9.99

**Table 2:** Indirect band gap energy of the PVA/PPy/KI/I_2_ electrolyte with different plasticizers.

Sample	Indirect Band Gap (eV)
PVA/PPy/EC/KI/I_2_	1.78
PVA/PPy/PC/KI/I_2_	1.90
PVA/PPy/GBL/KI/I_2_	2.19
PVA/PPy/DBP/KI/I_2_	2.26

**Table 3:** Electrical conductivity and activation energy of the PVA/PPy/KI/I_2_ electrolyte with different plasticizers.

Sample	Electrical Conductivity (S cm^−1^)	Activation Energy (kJ mol^−1^)
PVA/PPy/EC/KI/I_2_	0.496	0.863
PVA/PPy/PC/KI/I_2_	0.489	0.959
PVA/PPy/GBL/KI/I_2_	0.477	1.128
PVA/PPy/DBP/KI/I_2_	0.414	1.499

**Table 4:** EIS parameters for the PVA/PPy/KI/I_2_ electrolyte with various plasticizers.

Sample	R_s_ (Ω cm^2^)	R_ct1_ (Ω cm^2^)	R_ct2_ (Ω cm^2^)
PVA/PPy/EC/KI/I_2_	5.339	3.050	3. 879
PVA/PPy/PC/KI/I_2_	6.139	3.252	3.884
PVA/PPy/GBL/KI/I_2_	5.850	3.900	4.610
PVA/PPy/DBP/KI/I_2_	6.565	3.505	3.974

**Table 5:** Photo-voltaic parameters of fabricated DSSCs composed of different plasticized polymer electrolytes.

Sample	*V*_oc_ (mV)	*J*_sc_ (mAcm^−2^)	*FF*	Efficiency, *η* %
PVA/PPy/EC/KI/I_2_	0.714	11.097	0.530	4.19
PVA/PPy/PC/KI/I_2_	0.711	10.709	0.542	4.15
PVA/PPy/GBL/KI/I_2_	0.696	10.580	0.556	4.10
PVA/PPy/DBP/KI/I_2_	0.692	10.413	0.550	4.12

## Data Availability

Not applicable.
